# Social capital and fertility behaviors: a cross-sectional study in Iranian women health care workers

**DOI:** 10.1186/s12905-020-00943-5

**Published:** 2020-04-28

**Authors:** Mojgan Firouzbakht, Aram Tirgar, Karimollah Hajian-Tilaki, Fatemeh Bakouei, Mohammad Esmaeil Riahi, Maryam Nikpour

**Affiliations:** 1 Social Determinant of Health Research Center, Health Research Institute, Babol University of Medical Sciences, Ganjafrooz Street, Babol, Mazandaran Iran; 2 Department of Nursing- Midwifery, Islamic Azad University, Babol Branch, Babol, Iran; 3 Department of Biostatistics and Epidemiology, Babol University of Medical Sciences, Babol, Iran; 4 Infertility and Health Reproductive Research Center, Health Research Institute, & Department of Midwifery, School of Medicine, Babol University of Medical Sciences, Babol, I.R. Iran; 5 Department of Social Sciences, University of Mazandaran, Babolsar, Iran; 6 Social Determinant of Health Research Center, Health Research Institute, Babol University of Medical Sciences, Babol, Iran

**Keywords:** Childbirth behaviors, Social capital, Women, Health care workers, Iran

## Abstract

**Background:**

Fertility, in addition to the biomedical aspect, is phenomena of social, economic and demographic changes. Social network were considered for understanding fertility behaviors and changes in the levels of fertility. This study was conducted to investigate the relationship between social capital and childbirth behaviors in Iranian women employees.

**Methods:**

This cross-sectional study was conducted in 2017 on 536 women health care workers who randomly selected from health care setting Babol/Iran. Data were collected using demographic, childbearing behavior and social capital questionnaires. The SPSS-21 software was employed to analysis the data at a significant level of 0.05.

**Results:**

The results of the study showed that, there was significant relationship between number of pregnancy and social capital (*P* = 0.039). Furthermore, social capital has a significant relationship with the time of pregnancy (*P* = 0.043), the time of pregnancy in women with high social capital was observed to be relatively 30% shorter compare the women with low social capital.

**Conclusion:**

Social capital, as one of the important factors influencing childbirth behaviors, should be considered in population planning and policy making.

## Background

The move towards a contemporary lifestyle and the process of globalization has transformed the traditional family functions, which progressively has led to a change in the lifestyle of people and its various dimensions like reduction in birth rates [1, 2]. The rapid decline in the rate of fertility in Iran has been one of the most exceptional cases in the world over the past decades [3]. A decline of 70% in fertility rates over the past three decades [4], ranked Iran among the countries with a below- replacement level fertility rate (total fertility below 2.1 children) [5]. According to the World Bank’s predictions, with the continuous population decline in Iran, by 2025, population growth will fall below 1%, and the population structure of Iran will be completely made up of the aged [6].

Fertility is not just a biomedical dimension. It is a social phenomenon and it is subject to change with social, economic and demographic changes [7, 8]. Fertility behavior is a research area in which little is known about how meaning and subjective perceptions are created in interactions with relevant others and the way that they shape individuals’ behavior [9]. Over the last two decades, social networks were considered as one of the components of social capital to understand childbearing behaviors and changes in the level of fertility [10]. Social network is the communication of individuals in groups that can access resources and information [9]. New values were expanded through communication channels in social networks, and people became familiar with new perspectives about fertility; these new perspectives affect their childbirth behaviors [11]. In the study of Bernardi, social relationships had an influential role on fertility decisions, for instance, the impact of peer groups on fertility behavior in many aspects was more significant than the impact of the family [12]. Social interaction in social network creates social capital [13]. Social capital refers to resources obtained through social relationships [14]. It consists of trust, mutual understanding, shared values, and behaviors which connect people like a network, and facilitate achieving shared interests and goals [15]. Social capital makes it possible to access resources provided by social networking partners through direct (fair exchange of goods in the form of receiving and providing it in the short or long term) or indirect (trust, beliefs, and altruism) exchange [16]. The available resources of social network are similar to forms of insurance that could be used when needed [16, 17]. Childbearing has a variety of costs such as monetary cost, psychotic load, over load housework. The availability of resources that support the women to child care has a direct impact on childbearing. Also, some resources such as money, time, and ability to do work that help in improving or stabilizing the economic situation or the social status indirectly contribute to childbearing [17].

Limited empirical studies have been conducted on social capital and child-birth. Most studies have been conducted on social capital and childbirth in eastern European countries faced with a population crisis in the early twentieth centuries [10, 16–20] A study indicated that there was a strong supporting interaction with the desire to have a second child. The existence of a supportive environment creates a kind of social capital in relation to fertility. In this study, access to supportive resources at the individual’s level affected the fertility inclinations of people [17] . Balbo stated in their study that there is a non-linear relationship (U-shape) between receiving support and the desire to have a child in German men. Both the lack of access to support and over-coverage of circles (due to problems in their coordination) reduced the intention of having a second child among German men [20]. A study by Bernadi et al. also showed that social networking approaches can be used to explain the development of fertility inclinations [21].

The trend of population decline in Iran has been very disturbing in recent years. Although several studies have been carried out in this area, most studies focused on structural factors such as education and employment [22], and some study indicated on cognitive factors such as change in attitudes among women about the role of motherhood and wifehood [23]. In recent years, Iran has experienced a flourishing social capital and the neglect of same on the basis of sociological and demographic studies, respectively. Employed women working outdoors for many hours need a direct and indirect social network support for childbirth. The study of the behavior of childbirth in health care women, that appropriate knowledge and access to contraceptive methods, can show the relationship between social capital and women’s fertility behaviors more clearly. The aim of this study was to investigate the relationship between social capital and fertility behaviors in employed women.

## Methods

### Study setting and design

This cross-sectional study was conducted in 2017 in a group of employed women working in health care centers in Babol, northern Iran.

### Study population, sampling and inclusion criteria

The sample size in this study was estimated on the basis of the 0.18 effect size for social capital [24], 95% confidence level and 80% test power, five hundred subjects, and with estimated 20% drop out, was calculated as 600 people. Sampling was done through stratified random sampling. Initial, healthcare settings in Babol, Iran, were divided into the two main strata of hospitals and healthcare centers and then, eight healthcare centers and four hospitals were randomly selected by a draw from the strata. Finally, a convenience sample was selected from each center/hospital. The number of participants selected from each center/hospital was proportionate to the total number of its healthcare workers. Eligibility criteria in this study included married women with at least 1 year of employment in hospitals or health care center, absence of primary or secondary infertility precedent and any systemic disease affecting on fertility (such as advanced heart disease, malignancy precedent and chemotherapy healing). Exclusion criteria like withdrawal from participating in the study was also considered. In this study, the means of determining fertility behaviors were; the event of first pregnancy after marriage, and the total number of pregnancy.

### Data collection tools

The data for this study were collated with the use of three questionnaires. The first questionnaire measured the demographic and occupational characteristics of the participants. These characteristics included age, marriage age, marital status, educational level, husband’s education, husband’s job, satisfaction with economic situation, work experience and work shift. The second questionnaire measured the fertility behavior of the participants. The questionnaire included questions about pregnancy (number of pregnancies, number of childbirth), tendency to re-pregnancy (yes-no). The third questionnaire surveyed social capital impact using the Onyx and Bullen questionnaire [25]. The questionnaire consisted of 36 questions and covers various scopes which include the participation in social activities, communication with friends, communication with the family, etc. This questionnaire rated in four point Likert scale from 1 to 4. In this study, the mean score of social capital was considered as the cutoff point. A score higher than the cutoff point was a sign of high social capital and a lower score than the cut-off point meant a low social capital. Validity and reliability of this questionnaire were confirmed in Iran [26].

A total of 600 questionnaires were distributed among the participants and 536 questionnaires were identified for inclusion in the study and used for analysis (participation rate 89%).

### Data analysis

The data were analyzed with SPSS-21 software. To investigate the factors associated with the time of the first pregnancy after marriage, survival analysis, Kaplan-Meier method and Log-rank test were used. The variables affecting the number of pregnancies were examined using generalized linear model and using Poisson regression. The significance level in this study was considered as 0.05%.

### Results

The mean age of the participants in the study was 36.49 ± 7.78 years and the work experience was 11.24 ± 7.31 years (Median = 10). Eighty three percent (83%) were undergraduate respondents and 71.4% of them were nurses or midwives. A number of 60% of the respondents had expressed the idea of the child at the time of study, as well as in the time of marriage, two children. Forty five percent (45%) of the respondents had single child. A number of 14.4% of women were planning for re-pregnancy, and about 57% of them did not intend to become pregnancy even if they were supported by the government (Table 1).

**Table 1:** Demographics characteristics and fertility behaviors in women’s workers in Babol health centers. (*N* = 536)

Demographics characteristics		Mean ± SD
Age (year)		36.49 ± 7.78
Duration of work(year)		11.24 ± 7.31
Marriage age (year)		23.78 ± 3.70
First pregnancy after marriage (month)		22.97 ± 22.47
		N/%
Shift work	No	163 (30.4)
	Yes	373 (69.6)
Education	College under graduate	30 (4.7)
	Bachelor’s degree	445 (83)
	Master’s degree/Physician	61 (11.4)
Marital status	Married	510 (95.1)
	Separate	15 (2.8)
	Widow	11 (2.1)
Place of residence	Urban	45 (7.5)
	Rural	491 (91.6)
Husband education	College under graduate	24 (4.4)
	Bachelor’s degree	297 (55.4)
	Master’s degree/Physician	213 (40.2)
Economic satisfaction	Low	63 (11.7)
	Moderate	403 (75.1)
	High	70 (13.2)
Job	Nurses/midwife	383 (71.4)
	Health expert	127 (23.6)
	Physician	26 (5)
Number of pregnancy	0	65 (12.1)
	1	208 (38.6)
	2	176 (32.7)
	≥3	89 (16.6)
Number of child	0	59 (11)
	1	240 (45)
	2	161 (30)
	≥3	76 (14)
Ideal number of children in marriage time	0	26 (4.8)
	1	75 (13.9)
	2	288 (53.9)
	≥3	147 (27.4)
Ideal number of children in study time	0	–
	1	108 (20.1)
	2	320 (59.7)
	≥3	108 (20.1)
Intent to pregnancy in future	Yes	88 (14.4)
	No	312 (59.2)
	No Idea	132 (25.6)
Pregnancy if government support	Yes	121 (22.5)
	No	305 (56.9)
	No Idea	140 (26.1)

**Fig. 1:**
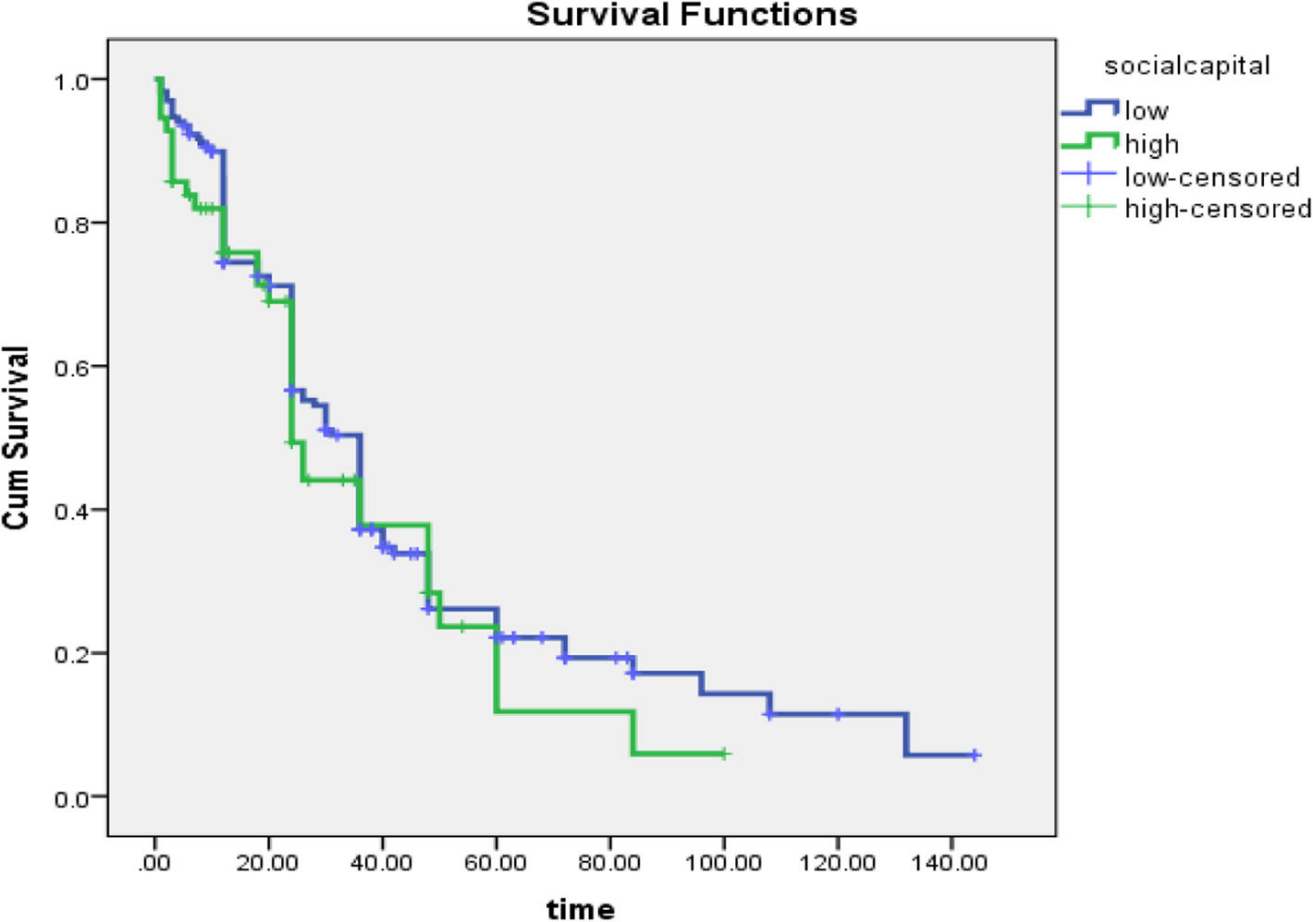
Survival time to event of pregnancy based on social capital in women’s workers in Babol health centers

The results of the Kaplan-Meier Survival Analysis and Log-Rank Test showed that the time of the first pregnancy after marriage with social capital possessed no significant relationship. Although, the mean difference in time of event of first pregnancy between two groups (social capital high vs. low), was evaluated with a Psychometrica web calculator [27], showed that, pregnancy occurred earlier in people with higher social capital (Cohen’s d = 1.143, CI 95%: 0.953–1.333) (Fig. 1).

**Fig. 2:**
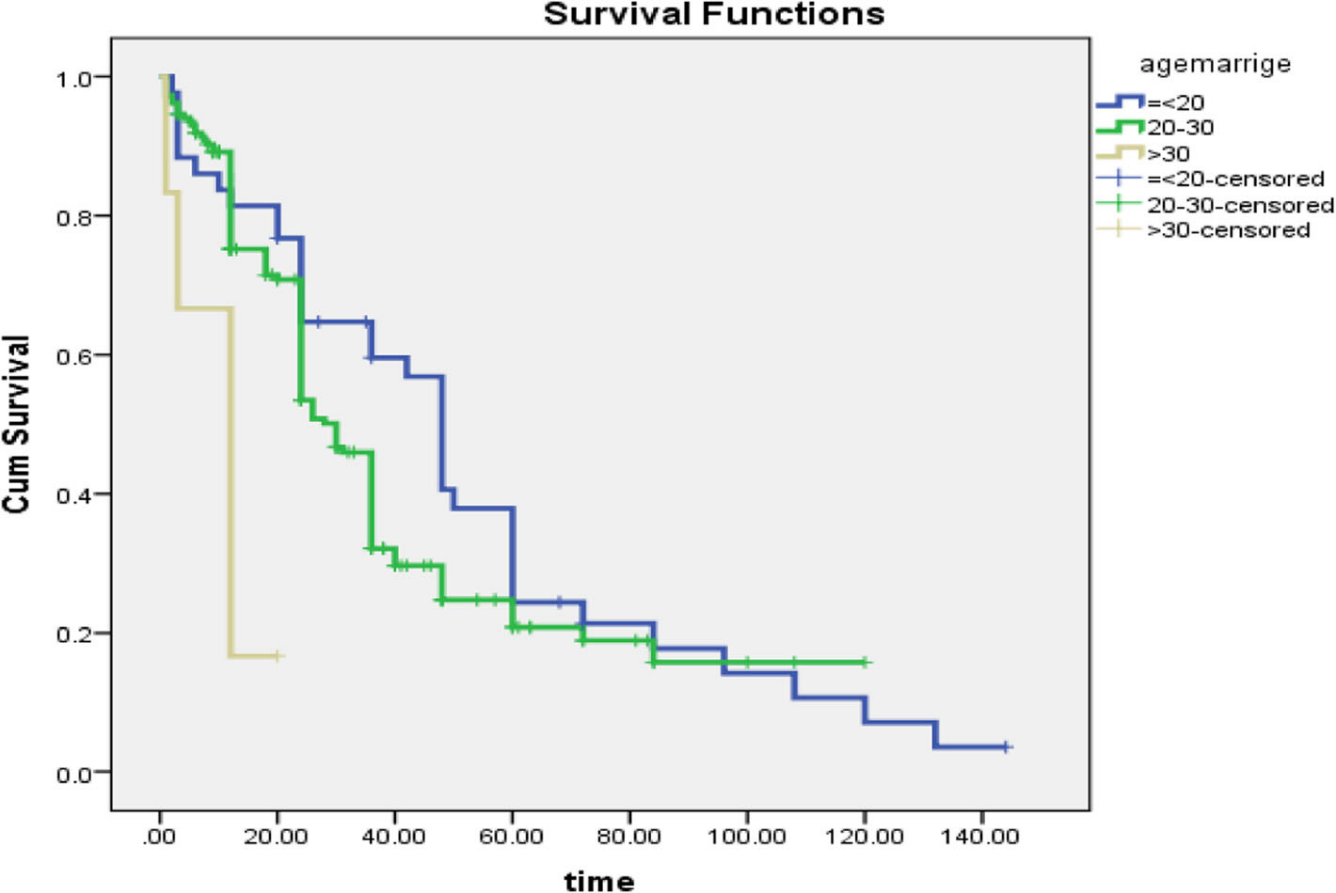
Survival time to event of pregnancy based on marriage age in women’s workers in Babol health centers

In this study, the time of the first pregnancy after marriage was significantly associated with the age of marriage (*P* < 0.001). In people with a higher marriage age, the event of pregnancy was earlier (Fig. 2).

**Fig. 3:**
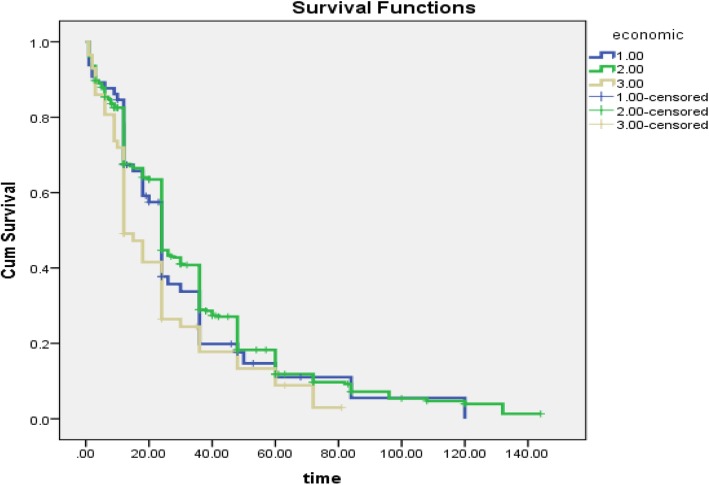
Survival time to event of pregnancy based on socio-economic in women’s workers in Babol health centers

Also, a significant relationship was detected between the time of occurrence of pregnancy and satisfaction of socio-economic status (*P* = 0.025), such that people with more satisfaction of the economic situation postponed their pregnancy (Fig. 3).The effect of the variables affecting the occurrence of the first pregnancy after marriage was investigated using Cox regression analysis. Social capital has a significant relationship with the time of pregnancy, the time of pregnancy in women with high social capital was observed to be relatively 30% shorter compare the women with low social capital (HR = 0.698, CI: 0.518–0.989, *P* = 0.043). Although, after adjusted with other variables, social capital has not significant relationship with the time of pregnancy (Table 2). Two variables; age of marriage and satisfaction of economic status with the time of first pregnancy, had a significant relationship. The time of event of pregnancy in people over the age of 30 years decreased by 47% compared to the women under the age of 30 years (HR = 0.532, CI: 0.326–0.868, *P* = 0.012), in other words, the women with the higher age of marriage, pregnancy occurred earlier. Moreover, people with low economic satisfaction had a higher probability of pregnancy than those with higher economic satisfaction, such that the time of event of pregnancy in people with low economic satisfaction was significantly 30% earlier than those with high economic satisfaction (HR = 0.732, CI: 0.545–0.998, *P* = 0.046).(Table 2).

**Table 2:** The Hazard Ratio (95% CI) for variables affected on event of first pregnancy in analysis of Cox regression model

	Model1				Model2			
	HR	CI 95%		P-Value	HR	CI 95%		*P*-Value
		Lower	upper			lower	upper	
Marriage age (> 30 vs. < 30)	0.541	0.331	0.885	0.014	0.532	0.326	0.868	.012
Economic satisfaction (high vs. low)	0.74	0.532	0.326	0.052	0.737	0.545	0.998	0.049
Social capital (high vs. low)	0.698	0.518	0.989	0.043				

Model1 The independent variables/ predictors were included Cox regression

Model2 The automatic forward Cox regression method

The results of Poisson regression indicated a significant relationship between the number of pregnancies and age (*P* < 0.001). Furthermore, there was a significant relationship between the number of pregnancy, marriage age, and social capital (*P* = 0.039) (Table 3).

**Table 3:** The Rate Ratio (95% CI) for variables affected on number of pregnancy in analysis of Poisson regression model

	RR	CI95%		P-Value
		Lower	Upper	
Age	1.047	1.032	1.048	<.001
Residence (Rural vs. Urban)	.819	.607	1.001	.051
Job (doctor vs. nurse)	.931	.668	1.299	.675
Social capital (high vs. low)	1.005	1.008	1.1	.039
Education				
college degree	1.182	813	1.697	.365
Bachelor’s degree	1.3301	.968	1.828	.079
Master’s degree. and higher ^Ɨ^				
Marriage age				
(< 20)	1.947	1.417	2.675	<.001
(20–30)	1.601	1.86	2.161	.002
(> 30) ^Ŧ^	1			

Ɨ: References, Ŧ: References

## Discussion

The aim of this study was to determine the relationship between fertility behaviors and social capital in employed women. In this study, social capital and childbirth behavior (number of pregnancies, number of children) showed a significant relationship. Childbirth alongside direct and indirect financial costs (such as physical exhaustion, extra work at home, and change in the relationship between parents), can affect parenting decisions in childbirth. Resources in networks can reduce these costs. The indirect participation of individuals in networks creates the opportunity for them to access the resources in the network without having to pay them back. Relationship between members of a family is an indirect participation. Family members are a significant source of support [16]. The family, in a number of ways, supports the participant by ensuring that other members of the family take care of their children; provide her with comfort, safety and security in their absence as well as coordinating their leisure activities with their leisure time. According to the social support theory, the social support benefits go to the individual that receives the support whether it persists permanently or not. Similarly, a woman who receives support and encouragement from relatives and friends to keep her child and to look forward to having another in the future tends to have a higher probability of having a child compared to woman who is deprived of such support and encouragement [28]. Social capital is influenced by the access possibility to material and non-material resources which affects fertility through the consolidation of the economic situation and social status [20].

In this study, women who had a higher age at marriage had the shorter interval between the first pregnancy and their marriage. These women try to be pregnant because of the awareness of the risks of becoming pregnant at middle age and avoiding its risk, as well as the desire to have a second child sooner [29]. Based on the theory of “rational action”, human beings act as a logical entity in a coherent and logical way. The intention to do a particular cause is expressed in an individual’s attitude and behavior. That is, if one wants to do something, it shows in his behavior [30]. According to this theory, the intention of fertility can greatly explain fertility behavior.

Although most women had expressed two children as the number of ideal children, however, the number of single children was higher among them. Razeghi Nasr Abad and Bagheri’s studies’ confirm the results of this study in relation to parents with single child [31, 32]. The intention to have the first child in a family is more affected by the desire to become a parent and under the influence of social pressures, while the desire to have a second child is influenced by the available economic and supportive resources that are needed for childbirth [33].

Reducing the number of children in comparison to their ideal number may be due to some of the phenomena including increased cost of living, increased cost of raising children and a changed attitude and mentality towards childbearing in the community. This result is in line with Bongaart’s theory that the high ideal number relative to the actual number of children is considered to be the phenomena of fertility transmission [34]. It seems that the reduction in the number of children in the employed women compared to their ideal number is indicative of fertility transmission to a lower level of the replacement level.

In this study, there was not a significant relationship between educational level and the number of children given birth to, but the number of children in women with high education was reduce. An increased level of education leads to an increase in the age of marriage [28, 35]. Several studies have shown the role of education at the time of marriage [33, 35]. The increase in the level of education in women leads to an increased opportunity cost of childbearing [36]. Gender preference decreases by reducing the functional differentiation between men and women. Consequently, a couple assumes that they have succession with only one child [37]. It is an attitude that leads to fertility under the substitution level. This view can remain as a norm among mentioned societies [38]. Based on the second demographic transition theory, the change in values ​​and ideology of individual development and self-realization and the importance of individual and social freedom were explained as the reasons for fertility change below substitution level. In fact, women will decide when they want to get pregnant, and the child will lead to the growth and development of their lives [39].

Another significant factor affecting the occurrence of pregnancy was the satisfaction of the economic situation. People with less satisfaction than the economic situation had an earlier pregnancy. Various studies have shown similar results to the findings of this study [40, 41]. Nevertheless, this relationship has not been observed in some studies [42]. Becker believes that educated parents who have high occupational levels and high cost- opportunities, as well as parents who are more likely to have easier access to pregnancy control tools because of their higher income, tend to have fewer children, and, conversely, low income parents have low paying jobs and no access to control methods for pregnancy [43, 44].

Based on the “supply and demand theory” and “opportunity cost theory”, the fertility behavior of individuals is affected by their economic behavior. In this theory, children are similar to consuming goods, which requires money and time. The number of children is determined on the basis of rational balance between having a child and other parent preferences. As income increases, the fertility rate increases and then the fertility rate decreases with increasing revenues. Therefore, the relationship between income and fertility is represented as a U-shaped curve [22].

This study is plagued by some limitations. It should be noted that the role of men was not investigated in this study. Because childbirth is a behavior affected by couples, it is necessary to examine the fertility studies of men for a more accurate examination. On the other hand, this study was done in a cross-sectional mode that does not show any causal relationship between variables. Moreover, in this study, the relationship between social capital and fertility behaviors was studied in general and the dimensions of social capital were not studied separately. But in any case, this study is the first study to examine the variables affecting fertility behaviors in working women who usually face more difficulties in the birth and maintenance of their children.

## Conclusion

The results of this study indicated the relationship between social capital and childbirth behaviors in employed women. The resources embedded in the social networks (as one of the component of social capital), decrease monetary and non- monetary costs of childbearing and effects on family’s decision on childbearing. Hence, one of the ways to cope with the decline in population growth in Iran can be the promotion of social capital.

## Data Availability

The datasets analyzed during the current study are available from the corresponding author on reasonable request.

## Abbreviations

SPSS: Statistical Package for the Social Sciences
SD: Standard Deviation
N: Number
RR: Relative Rate
HR: Hazard Ratio
CI: Confidence Interval
